# Dioxin and Related Compound Detection: Perspectives for Optical Monitoring

**DOI:** 10.3390/ijms20112671

**Published:** 2019-05-30

**Authors:** Barbara Patrizi, Mario Siciliani de Cumis, Silvia Viciani, Francesco D’Amato

**Affiliations:** 1 National Institute of Optics-National Research Council (INO-CNR), Via Madonna del Piano, 10, 50019 Sesto Fiorentino, Italy; barbara.patrizi@ino.cnr.it (B.P.); silvia.viciani@ino.cnr.it (S.V.); francesco.damato@ino.cnr.it (F.D.); 2 European Laboratory for Non-Linear Spectroscopy (LENS), Via Nello Carrara n. 1, 50019 Sesto Fiorentino, Italy; 3 Italian Space Agency, Contrada Terlecchia snc, 75100 Matera, Italy

**Keywords:** dioxins, 2,3,7,8-TCDD, PCBs, POPs, optical detection, environmental xenobiotics, dioxin bio-monitoring, dioxin detection

## Abstract

Dioxins and related compounds are environmental xenobiotics that are dangerous to human life, due to the accumulation and persistence in the environment and in the food chain. Cancer, reproductive and developmental issues, and damage to the immune system and endocrine system are only a few examples of the impact of such substances in everyday life. For these reasons, it is fundamental to detect and monitor these molecules in biological samples. The consolidated technique for analytical evaluation is gas chromatography combined with high-resolution mass spectrometry. Nowadays, the development of mid-infrared optical components like broadband laser sources, optical frequency combs, high performance Fourier-transform infrared spectroscopy, and plasmonic sensors open the way to new techniques for detection and real time monitoring of these organic pollutants in gaseous or liquid phase, with sufficient sensitivity and selectivity, and in short time periods. In this review, we report the latest techniques for the detection of dioxins, furans and related compounds based on optical and spectroscopic methods, looking at future perspectives.

## 1. Introduction

Dioxins are among the most toxic and persistent organic pollutants (POPs). They are principally released into the atmosphere as undesired products of various combustion and industrial processes [1] such as incineration of municipal and medical solid waste, backyard waste burning, paper and wood pulp chlorine bleaching, coal fired power plants, and also from natural processes like forest fires. Furthermore, these pollutants also occur as contaminants in several pesticides, herbicides, and fungicides [2].

When accidently released in the various environmental matrices, like soil and water, dioxins accumulate in plants and animal tissues, until they reach human tissues where accumulate at higher and higher concentrations, mainly in the fatty tissues, through food chain biomagnifications processes.

**Figure 1:**
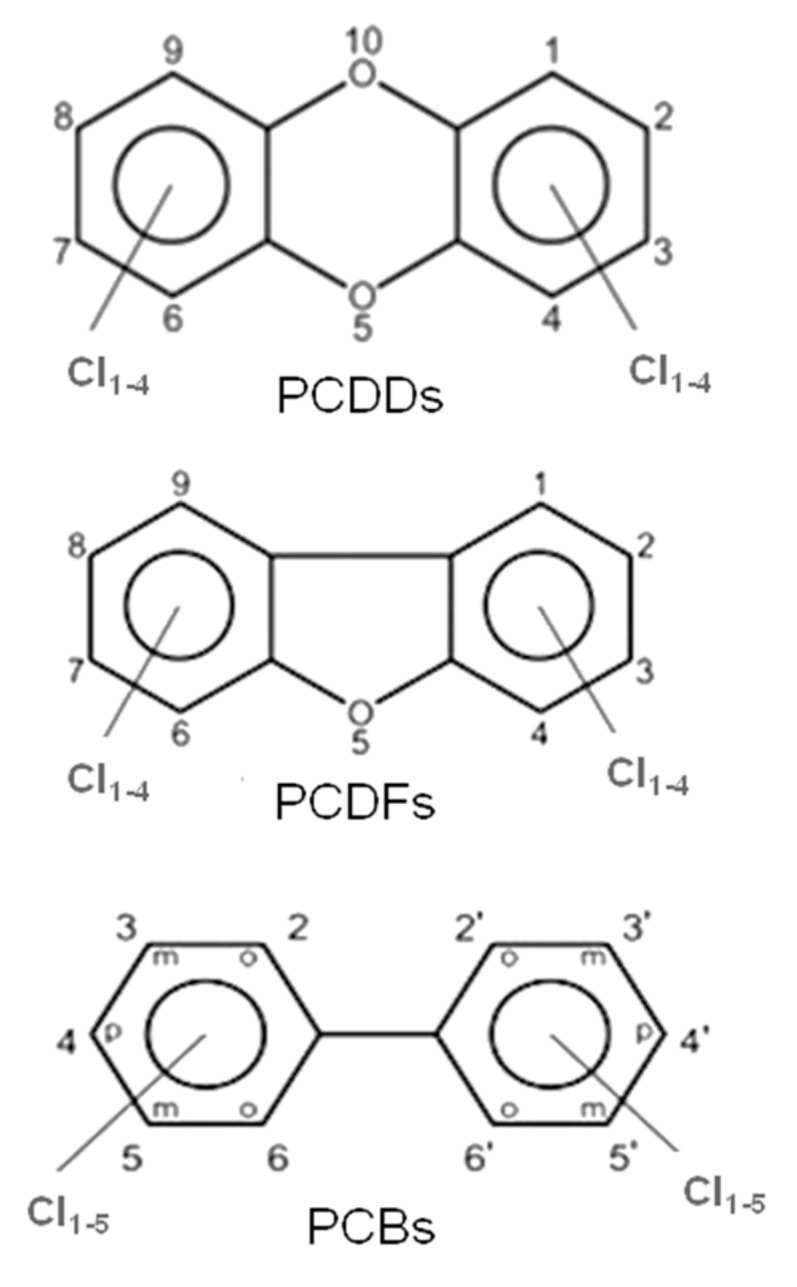
General molecular structures of chlorinated dioxins (PCDDs), furans (PCDFs) and biphenyls (PCBs).

These halogenated organic POPs mainly belong to three families of molecules, i.e., polychlorinated dibenzo-p-dioxins (PCDDs), polychlorinated dibenzofurans (PCDFs) and polychlorinated biphenyls (PCBs); their molecular structures are presented in Figure 1.

All these compounds together with Polychlorinated Diphenyl Ethers (PCDEs) belong to the main family of Polyhalogenated Aromatic Compounds (PHAs). PCBs and PCDEs are industrial compounds or by-products, mainly present as impurities in chlorophenol preparations. These two classes of POPs have been detected in the environment and especially in chemical-waste dumpsites. PCBs, in particular, are synthetic PHAs used as industrial reagents in the past. Because they persist for long times in both the environmental matrices and in tissues of living organisms, their production and utilization were discontinued.

PCDDs and PCDFs have two benzene rings connected by one (furans) or two (dioxins) oxygen atoms. The two benzene rings can bind from one to eight chlorine atoms, generating a wide family of congeners (75 PCDD congeners and 135 PCDF congeners) whose molecular reactivity toward cellular targets can change dramatically, determining different levels of toxicity. Within these 210 congeners, the 2,3,7,8-tetrachlorinated dibenzo dioxins (TCDD) species have been classified as the most toxic. Toxicity drastically decreases when non-lateral chlorines are present or when lateral chlorines are removed from the two aromatic rings [3]. Laterally chlorinated dioxins and especially TCDD show the highest affinity toward the AhR (Aryl hydrocarbon Receptor) receptor, a signal transducer protein which is responsible of the biological effects cascade following the xenobiotic–receptor interaction [4,5,6]. There are seven laterally chlorinated PCDDs and ten laterally chlorinated PCDFs. The toxicity equivalent factor (TEF) [7] expresses the toxicity of dioxins, furans and PCBs in terms of the most toxic congeners, i.e., the 2,3,7,8-TCDD and 1,2,3,7,8-pentachlorodibenzo-p-dioxin, whose TEF value has been set to 1. It must be taken into account that dioxins are extremely toxic molecules; indeed, they can cause cancer, reproductive and developmental aberrations, immune system damages, and can deeply interfere with the endocrine system [8,9,10,11].

In this regard, it is important to emphasize that the half-life of TCDD in humans is very long; it has been estimated to be in the range between 7.1 [12] and 11.3 years [13].

Similar to dioxins and furans, the number of chlorine atoms (from 1 to 10) and their positions in PCBs give rise to 209 congeners (see Figure 1).

In particular, ortho-PCBs are characterized by two chlorine atoms in ortho positions, while mono-ortho-PCBs (i.e., PCBs 105, 114, 118, 123, 156, 157, 167, and 189) are characterized by one chlorine atom in ortho position. On the other hand, in non-ortho-PCBs (i.e., PCBs 77, 81, 126, and 169) non-ortho positions are occupied by chlorine atoms. Both non-ortho and mono-ortho PCBs have coplanar structures that make them more toxic since they bind with higher affinity the AhR receptor. For this reason non-ortho and mono-ortho PCBs have been classified with different values of TEF [14], likewise dioxins and furans.

In particular, PCB 77 (3,3′4,4′ tetrachlorobiphenyl) is able, like TCDD, to bind strongly to the AhR receptor leading to the activation of the expression of genes involved in polycyclic aromatic hydrocarbon metabolism and detoxification [6,15]. Prolonged exposure to PCBs leads to immune system suppression increasing the risk of diseases developing. Both non-ortho and mono-ortho PCBs congeners are cancer promoters and enhance the effects of other carcinogens. PCBs are endocrine disruptors, altering thyroid and reproductive function in males and females. These endocrine alterations increase the risk of developing of other cardiovascular diseases and diabetes [16].

Due to their high toxicity and environmental persistence, many efforts for real-time monitoring of these organic pollutants from the principal emission sources have been recently realized [17,18,19,20,21]. Unfortunately, the objective of constant monitoring of the potential sources of emission and the possibility of recognizing each single congener in a complex mixture have not yet been fulfilled.

Moreover, it is also very important to detect dioxins and related compounds in environmental matrices such as soil, water, air and so on. PHAs are ubiquitous in the environment, they have been found in aqueous (mainly surface water, plant oils and petroleum products), air and solid samples such as sediments, soils and wastewater sludge. In this regard, very often it is of fundamental importance to isolate PHAs not only from environmental matrices, but also from biological tissues and fluids, by extraction and clean-up procedures. The clean-up step allows eliminating the matrix effects and pre-concentrates the analytes for instrumental quantitative detection. In the last years several techniques were developed and implemented for environmental samples clean-up, employing different kind of adsorbents materials such as silica gel and alumina, mesoporous organic silica, mesoporous silica nanoparticles, different nanoparticles and nanotubes functionalized at their surface, and molecularly imprinted polymers for more efficient adsorption capacity [22,23].

Then, the analytes must be extracted by using a suitable solvent, in order to proceed with the appropriate instrumental detection.

In the last years, several instrumental automated extraction techniques for rapid sample processing have been developed. Some of these techniques are supercritical fluid extraction (SFE), microwave-assisted extraction (MAE), pressurized liquid extraction [24], and solid-phase extraction (SPE) [25,26,27].

Due to their high resolution, high sensitivity and selectivity liquid and gas chromatographies (LC and GC) are the most commonly employed analytical methods for the qualitative and quantitative determination of POPs in environmental and biological samples. In particular, High Performance Liquid Chromatography (HPLC) coupled with UV/fluorescence detection, and Gas Chromatography coupled to Mass Spectrometry (GC/MS) has shown to be the best technique amongst others. GC/MS is a reference analytical method because provides the advantages of congener identification using both retention time and mass spectrum [28,29,30]. Furthermore, GC/MS provides the required high sensitivity for quantification in the Selected Ion Monitoring (SIM) mode.

Even thought Liquid Chromatography coupled to Mass Spectrometry (LC/MS) offers similar advantages, it does not supply a specific interface appropriate for the separation of all the PHAs at the same time [28,31].

The possibility to use new generation materials for the separation and clean-up and to automate instrumental extraction of pollutants from environmental and biologic samples will allow new optical detection methods to become more selective and sensitive and to achieve real time and fast monitoring of chlorinated organic compounds.

## 2. Optical Monitoring of Dioxins and Related Compounds, a Screening of Old and New Mid-IR Light Sources and Techniques

Optical monitoring offers many advantages with respect to the cited techniques like chromatography or high-resolution mass spectrometry in terms of costs, destructive sampling and fast detection. Optical techniques can be divided into two main classes: (1) those which employ wide wavelength range sources and (2) those which use narrow wavelength range sources. Let us discuss them separately.

### 2.1. Wide Wavelength Range Sources

The use of Mid-IR radiation to detect PCDD/Fs and related compounds allows discriminating between congeners, since their own fingerprints in this spectral region characterize these molecules.

**Figure 2:**
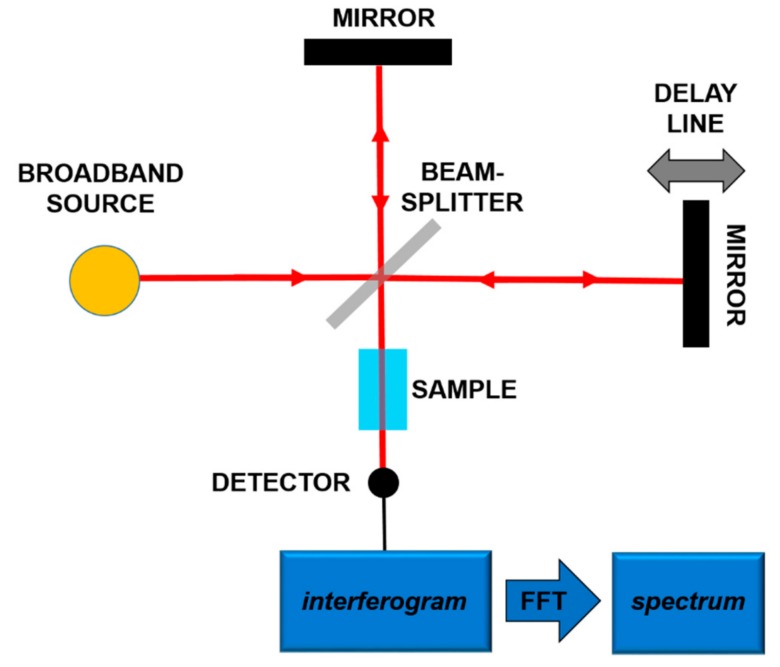
Principles of the Fourier-Transform Infrared Spectroscopy (FT-IR).

The most frequent use of the absorption spectroscopy in Mid-IR lies in the identification of substances through their characteristic molecular vibrations. Furans and dioxins, and in general, molecules with a similar structure, are not in gaseous phase at room temperature, and high temperatures (more than 550 °C) are needed to perform gaseous spectroscopy. However, in order to work at room temperature, it is possible to carry out spectroscopy in liquid phase, dissolving the target molecules in a Mid-IR transparent solution [18,19]. In both gaseous and liquid spectroscopy, the detected absorption peaks are tens of cm^−1^ wide. In order to detect these bands, techniques and components spanning a wide frequency range are needed. In this framework, a consolidated technique is Fourier-Transform Infrared Spectroscopy (FT-IR) [32]. In this scheme, sketched in Figure 2, using a broadband source, typically a lamp, an interferometric pattern is recorded from the light interacting with the sample. By using the Fourier Transform approach, it is possible to reconstruct the absorption spectrum of the substance under analysis.

**Figure 3:**
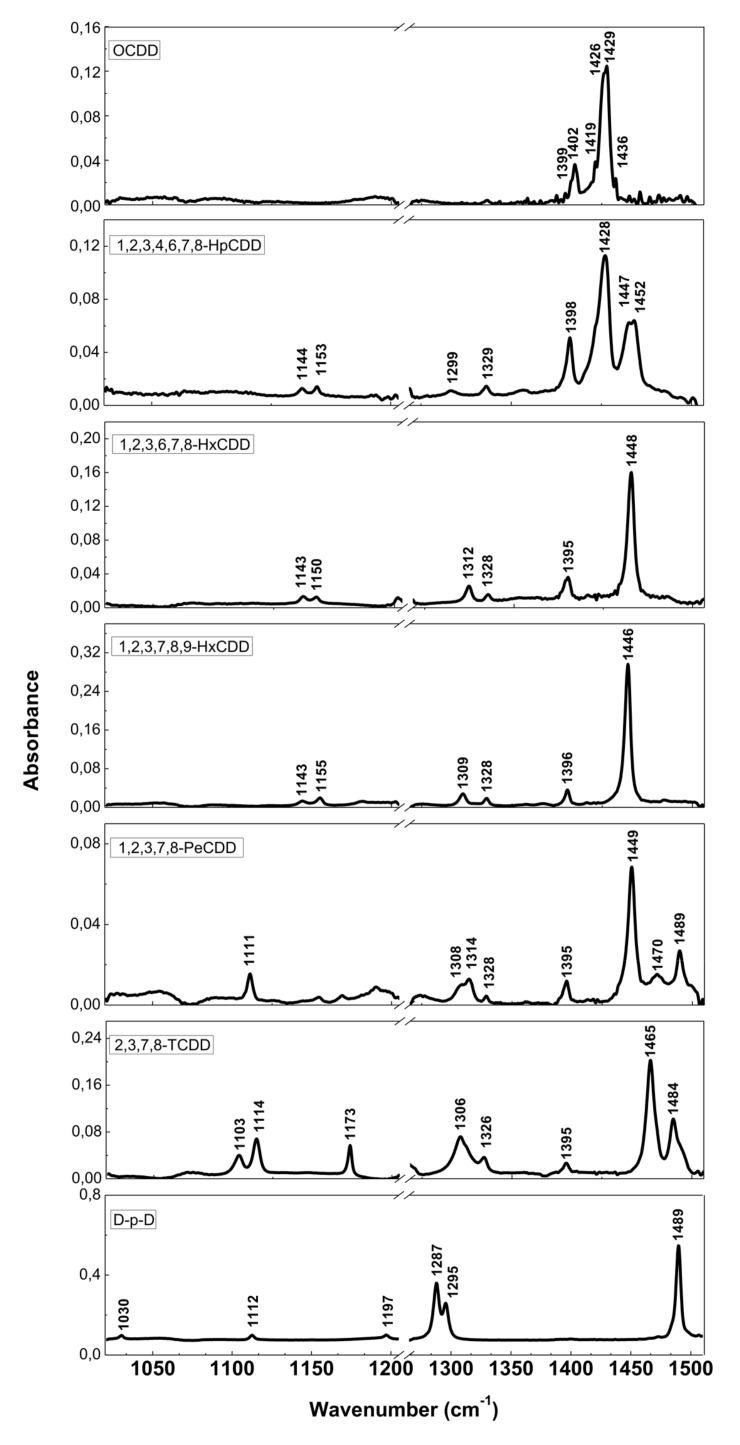
FT-IR spectra of PCDDs and dibenzo-p-dioxin (D-p-D). Reprinted with permission from [19], copyright 2014.

**Figure 4:**
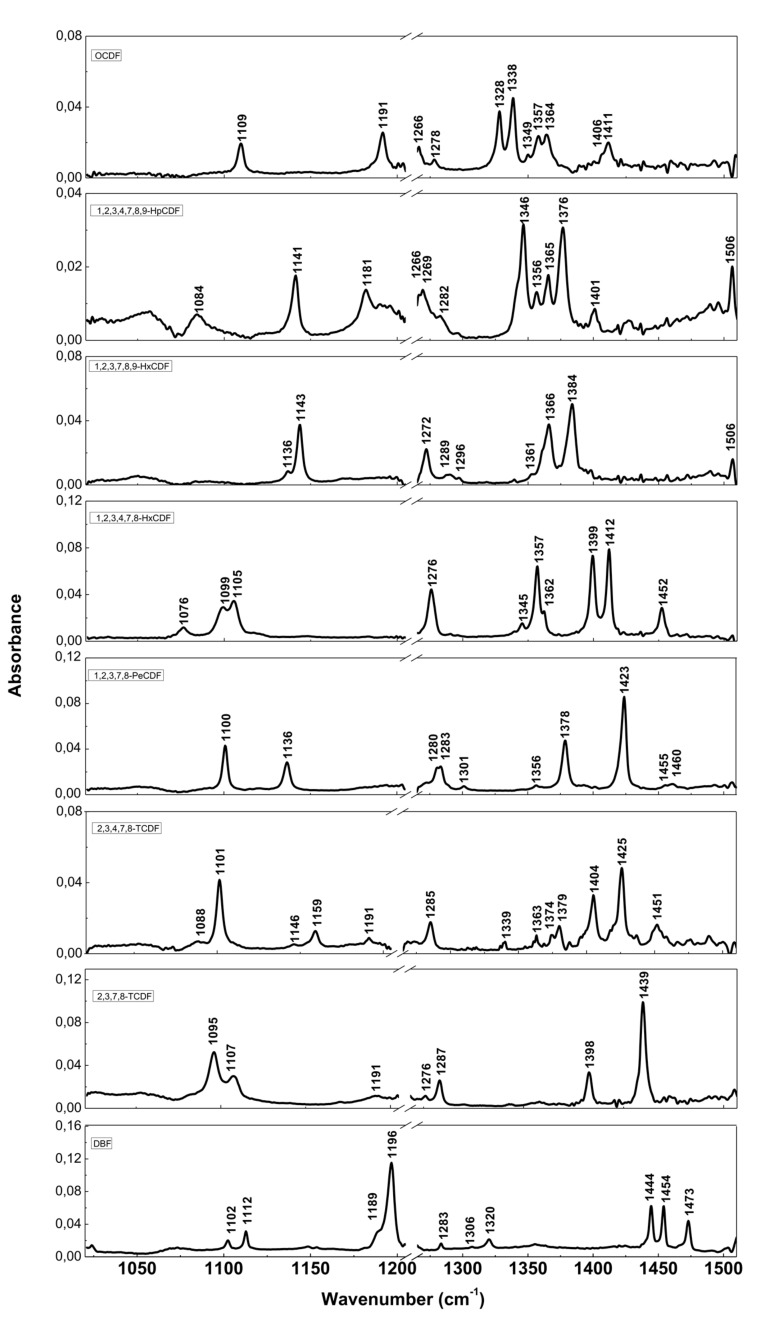
FT-IR spectra of PCDFs and dibenzofuran (DBF). Reprinted with permission from [19], copyright 2014.

The resolution of commercial instruments is better than 1 cm^−1^. This technique has been used for measuring dioxins, furans and related chlorinated organic molecules. In Figure 3 and Figure 4, we report the infrared spectra of 13 of the 17 most toxic congeners of dioxins dissolved in carbon tetrachloride (CCl4) [19]. In Table 1 and Table 2, the characteristic vibrational frequencies of PCDDs and PCDFs, obtained by FT-IR in condensed phase [19] and other techniques, i.e., the GC/MI/FT-IR [33] and Vapor-Phase FT-IR [34], are presented and compared. In Table 1 and Table 2, each frequency range is characterized by different IR active modes that represent the fingerprints of each single PCDD/Fs congener.

These vibrational frequencies are influenced by chlorine substitution patterns and thus allow us to discriminate between different molecules and congeners, and also to analyze mixtures of these compounds [19].

Although this method is robust and consolidated, FT-IR performance is limited by the brilliance of the light source used.

In the last decades the development of powerful infrared sources like Interband Cascade Lasers (ICL), Quantum Cascade Laser (QCL), QCL combs, optical frequency combs, Non-Linear sources [35,36,37,38,39,40,41] paved the way for the improvement of the performances of classical techniques, or innovative detection scheme. In particular, these new sources could be very suitable for detecting complex organic molecules (as dioxins, furans, hydrocarbons, proteins) which present absorption spectra in the mid-infrared with absorption profiles width of ten or more wavenumbers. For simplicity, in the following, we refer to molecules with these characteristics as “complex molecules” [42].

Typically, narrow laser sources are used for spectroscopy of simple molecules in gas phase, like CO2, H2S, CO, HF with a large variety of experimental setups [43,44,45,46,47,48,49].

**Figure 5:**
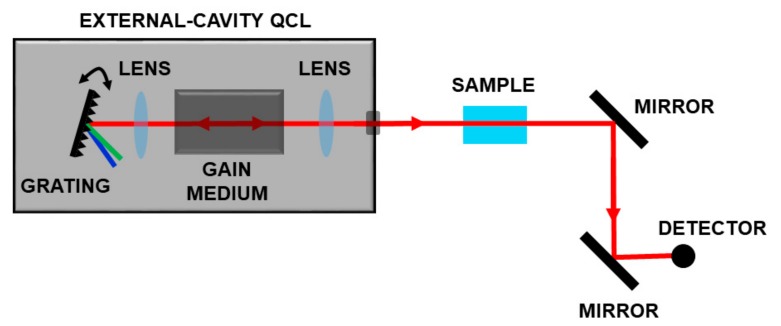
Spectroscopic set-up based on a broadband tunable QCL. By moving a grating, it is possible to select the operation wavelength on a very large frequency range.

In the last ten years, the availability of broadband tunable QCLs in the range 4–12 µm enabled the development of optical schemes for measuring complex molecules. These sources, based on a moving external grating, which closes the laser cavity, allow wavelength selection and high gain for the active media on a very large frequency range as sketched in Figure 5.

The application of such schemes spans from health to security, from imaging to environmental monitoring [50,51,52,53,54,55].

Using a couple of broadly tunable external cavity QCLs (EC-QCLs), the detection of furans and dioxins dissolved in CCl_4_ was demonstrated [18] in the range 1205–1310 cm^−1^ and 1335–1590 cm^−1^. The measurement time spanned from few minutes to 15 minutes. Nowadays, the technological development of this kind of source allows the same measurement using only one laser frame equipped with 3 or 4 active media [56] with tuning performances up to 1000 cm^−1^, reaching speeds up to thousands cm^−1^/s in particular cases. This enables very fast measurements on very large frequency ranges, with frequency resolution strongly dependent on the acquisition system. Generally, a fast scan corresponds to low frequency resolution. When using such systems for liquid spectroscopy, the accuracy of measurements depends on many parameters, and above all on the sample itself. For example in the biomedical field, Lendl and co-workers successfully used EC-QCLs for detection of glucose and lactate in aqueous phase, showing the usability of such laser sources for in-vivo and in-vitro bio fluid solutions [51,57,58].

EC-QCLs have already been extensively used in biological and fundamental chemistry studies involving peptide, breath test, kinetics reactions and DNA research [59,60,61].

In addiction such lasers, due to their high optical power, could be used for infrared imaging with tens of different approaches: Chemical Imaging, Photo-thermal imaging, holography, microscopy, etc. [62,63,64,65,66].

However, EC-QCLs cannot be used when it is necessary to observe and resolve fine absorption structures, narrower than the laser line (which is typically at least tens of MHz), or when the scan rate is still too slow compared to the experimental time scale.

A different approach in broadband spectroscopy is provided by Optical Frequency Combs (OFCs) laser systems. Two main features of such lasers are the characteristic equally spaced comb spectrum and the very narrow linewidth. Indeed they are largely used for measuring absorptions of simple molecules in the visible and near infrared using different schemes: Comb assisted Spectroscopy, Direct Comb Spectroscopy, Dual Comb Spectroscopy [35,67,68,69,70,71].

**Figure 6:**
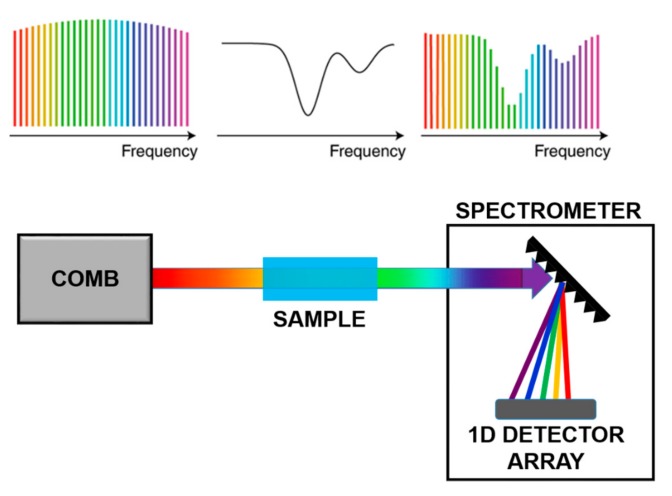
Scheme of Direct Comb Spectroscopy. Adapted with permission from [68], copyright 2019.

In direct comb spectroscopy, as shown in Figure 6, each tooth interacts with the sample and the absorption is measured tooth by tooth, using a dispersive system on a single detector or a 1-D or 2-D detector arrays. This technique enables the reconstruction of fine structure absorption profiles with large accuracy. However, to apply this approach to organic molecules, the comb spectrum has to be translated in the Mid-IR via Non-linear processes.

**Figure 7:**
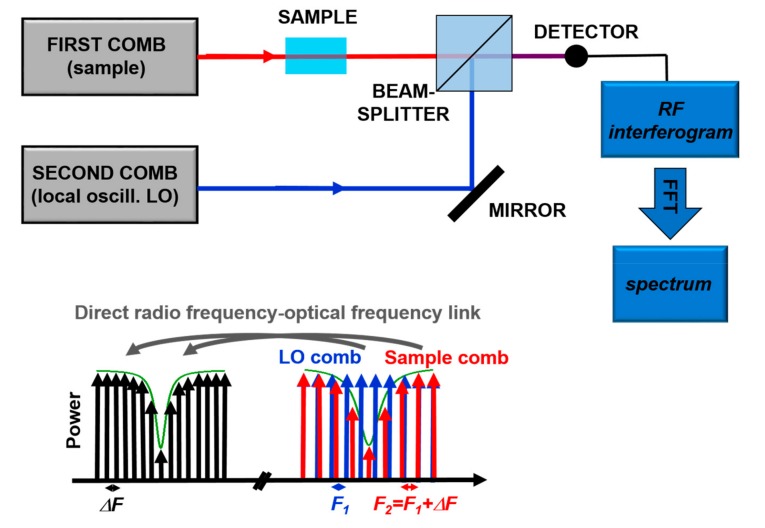
Principles of Dual-Comb Spectroscopy. Adapted with permission from [70], copyright 2014.

Recently, Dual Comb Spectroscopy is proving to be a very powerful technique. It is based, as sketched in Figure 7, on the detection of the interference between two combs with slightly different tooth spacing in the frequency-domain. The detected heterodyne beat signal consists of a comb in the Radio-Frequency (RF) domain, so that the absorption signal is translated from the optical frequencies to the “more easy to measure” RF spectrum. From the Fourier transform of the acquired signal it is possible to reconstruct entire absorption bands, like in FT spectroscopy but without moving components, with the frequency resolution and accuracy of OFCs. Until recently, this type of technique was limited to the spectral range covered by OFCs, typically in the Near IR. However, the invention of QCL-combs pushed the development of such technique also in the mid-IR with some commercial instrument available on the market. The strong point of Dual Comb Spectroscopy is the combination of accuracy, very fast measurement time, and high brilliance of the source [70]. This technique was successfully demonstrated in a protein reaction study in the region around 8 µm [72], thus paving the way for the non-invasive analysis in biological context.

In Table 3, we report a summary of the described optical detection techniques in the 1000–1500 cm^−1^ region. The first two techniques have already been used to analyze PCDD/Fs [18], while the last two techniques have the potentiality to be used for detecting dioxins and furans, but they have not yet been applied for this purpose. Their potentiality is demonstrated by the fact that these techniques were used to measure organic molecules [56,72] with similar absorption characteristics. The most performing technique is the dual comb spectroscopy based on QCL combs. In this case, the price to pay is a limited spectral coverage and a very high cost. The cheapest instrument is the consolidated FT-IR spectrometer, but the low optical power represents a limit for analysis in thick samples or cells (hundreds microns). The EC-QCLs, due to their high power, are suitable for imaging, spectroscopy in longer measurement cells (few millimeters in liquid phase), and for slow measurements.

### 2.2. Narrow Wavelength Range Sources

Mid-IR Laser technology is nowadays gaining interest due to new device development. However, sometimes chemical analysis and biological assays need a fast response, not necessarily quantitative but at least indicative of the presence of a toxic molecule. Therefore, a class of sensors characterized by cost-effectiveness, ready for real-time and on-site analysis is highly desirable. In many cases, sensors might be used for the determination of dioxins and dioxin like-PCBs throughout the environment and the food chain, involving various types of specimens such as water, air, soil, food, animal tissues and so on. So far, we compared different techniques based on wide spectrum sources. However, there are other classes of optical sensors based on lasers with limited tunability (a few nm at most) and possibly cheaper. One of them takes advantage of plasmonic resonances, and is able to detect complex organic molecules [73,74,75,76,77,78]. These sensors would deserve a broader discussion, so we refer also to other review articles and books present in the literature [73,79].

**Figure 8:**
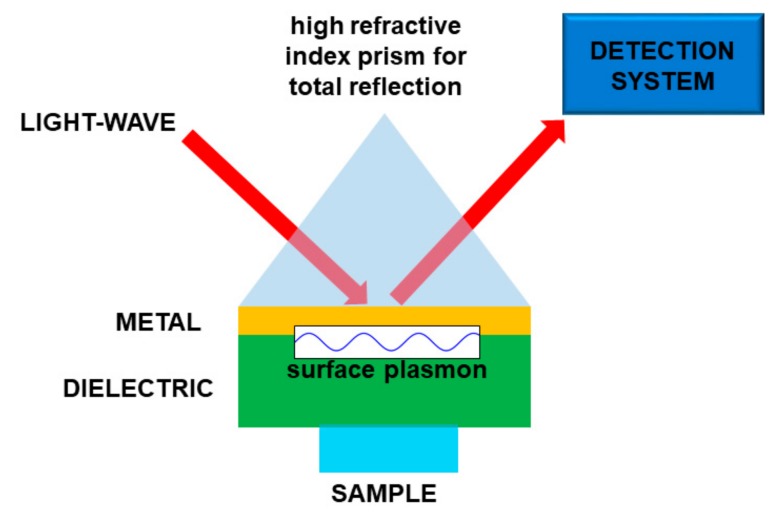
Sketch of a plasmonic sensor. In this particular case the light is used to “read” the sensor. Molecule “detection” is performed by means of the interaction between the sample and a material supporting the surface plasmons.

Basically, the interaction between the target molecule and the structure (nanoparticles, nanomaterial, fiber sensors), provided with plasmonic resonances, produces a relevant effect in absorption, fluorescence enhancement, or frequency shifting of a laser beam. In the latter case, when the molecule is on the plasmonic structure, the change of physical conditions at the interface produces some changes in the plasmon coupling, namely, an easily detectable resonance shift. In this way it is possible to obtain a very sensitive sensor, tailored onto a specific target molecule or molecular class. In most cases, the plasmonic sensor needs to be functionalized to enable the sticking of the molecule to the structure, so making the detection possible. A scheme of a kind of plasmonic sensor is reported in Figure 8.

In the literature there are some examples of plasmonic sensors for measuring dioxins, dioxin precursors, polychlorinated biphenyls and atrazine [80,81]. The strong point of these sensors is the very high selectivity and sensitivity [81], down to concentrations of the order of ng/mL. In some cases, single molecule detection could be achieved. It is fundamental specifying that this kind of plasmonic sensor detects the molecule sticking on the surface, so the chemical selectivity is due essentially to the selectivity of the functionalized material. For example, if this material is able to capture all the molecules of dioxin and furan families, the sensor could detect also a single molecule but it would not discriminate between the most toxic 2,3,7,8 TCDD and the non-toxic DBF. For evident reasons, plasmonic sensors could suffer polluting phenomena, so they may have to be cleaned or replaced. This could not be a real problem, as using some classes of plasmonic structure or functionalizing materials, plasmonic sensors could be very cheap.

Similar mechanisms of operation are common to other kind of sensors based on Surface-Enhanced Raman Scattering (SERS), [82,83,84,85,86,87], fluorescence quenching and enhancement [88] and surface photo voltage [89]. Raman spectroscopy is a well-established technique, based on the inelastic scattering of light. Starting from a single-frequency laser excitation it is possible to get from the target molecule one or more emissions at lower (Stokes) or higher (anti-Stokes) frequencies. The frequency differences, with respect to the pumping laser, are typical of each target, so allowing selectivity. Usually the Raman signal deriving from a single layer of molecules is very low. When plasmon resonance is involved, the effect rises by a factor up to 108, and this allows single molecule detection. For many years, the poor reproducibility of the metal coatings needed for SERS limited the applications of this technique. Nevertheless, since a couple of decades ago, this technological issue has been overcome and applications became possible. In the literature, it is possible to find examples of detection of dioxins [82] and of other molecules, like pesticides [90].

SERS is one of the most employed spectroscopic techniques for ultrasensitive detection in chemistry and biology. One of the most important requirements in this field is the design of advanced materials capable of generating high-quality SERS signals. Zhu et al. [85] used Ag nanoplate-assembled films as SERS substrates for the rapid detection of PCB-77. The authors obtained a detection limit of about 10^−7^ M (mol/L) and found a linear dependence between the logarithmic concentrations of PCB-77 and the fingerprint peaks intensities. The authors used the same Ag nanoplate-assembled also to distinguish the characteristic peaks of different PCBs congeners in mixed solutions. Abalde-Cela and co-workers [82] obtained the SERS spectrum of 2-benzoyldibenzo-p-dioxin at a concentration lower than 10^−8^ M (mol/L), a detection level that matches current immunological methods. They used a SERS substrate based on thin films made by exponential layer-by-layer, infiltrated with silver nanoparticles. The authors found a characteristic vibrational pattern characterized by bands at 1392 (ring stretching) and 930 cm^−1^ (ring breathing), and smaller intensity bands at 1641 (CO stretching), 1605 and 1445 (ring stretching), 1296 (CO stretching), 852 (CH wagging), and 791 (ring deformation) cm^−1^.

These sensors represent a rapid, direct, and ultrasensitive detection method for environmental pollutants from different matrices.

Wang and co-workers [88] reported on the use of a sensitive and selective fluorescent membrane for rapid detection of trace 2,2′,4,5,5′-pentachlorinated biphenyl (PCB101). The fluorophore phenyl isothiocyanate (PICT) was immobilized onto porous anodic aluminium oxide membrane. The fluorescence of this membrane is enhanced after adding the analyte PCB101 into the membrane, due to the interaction between the fluorophore PITC and the analyte PCB101. The fluorescence intensity increased with the PCB101 concentration in the low range below 1 ppm, and the membrane showed good selectivity.

## 3. Conclusions and Future Perspectives

Following these premises, optical monitoring of these organic molecules in liquid phase with high time resolution, in vitro (with minimal sample consumption) or even in vivo, is not so far from being achieved.

Nowadays, the development of photonics has already provided us with some tools, i.e., laser sources such as OFC, ICL, QCL, ICL- and QCL-comb, non-linear sources. Furthermore, other new mid-IR components such as waveguides, micro fluidic/waveguides, detectors [91,92] are under development and implementation, thus paving the way toward compact instruments for in vivo measurements of dioxins and related toxic compounds.

On the other hand, plasmonic sensors represent a compact alternative to Mid-IR sensors for non-invasive analysis or in-vivo measures, although their selectivity is strictly dependent on the material used to selectively capture the molecule of interest, and they could suffer surface pollution with a consequent performance reduction.

## Figures and Tables

**Table 1:** Principal FT-IR bands of PCDDs. Reprinted with permission from [19], copyright 2014.

PCDD Congener	Mode	Frequency Range (1020–1205 and 1265–1510 cm^−1^) GC/MI/FT-IR [33]	Frequency Range (1020–1205 and 1265–1510 cm^−1^) Vapor-Phase FT-IR [34]	Frequency Range (1020–1205 and 1265–1510 cm^−1^) Condensed-Phase FT-IR [19]
dibenzo-*p*-dioxin	C=C aromatic ring skeletal vibrations		1489	1489
			1390	
	C-O-C asymmetric stretch			1295
			1290	1287
	C-C-C tri-ring bending			1197
			1117	
	Ring breathing		1026	1112
				1030
2,3,7,8-TCDD	C=C aromatic ring skeletal vibrations	1495		
		1488	1489	1484
		1470	1465	1465
			1391	1395
	C-O-C asymmetric stretch	1330	1321	1326
		1313		
		1306	1306	1306
	C-C-C tri-ring bending	1176	1173	1173
	Ring breathing	1117	1114	1114
		1106	1103	1103
1,2,3,7,8-PeCDD	C=C aromatic ring skeletal vibrations	1493		1489
		1475	1471	1470
		1465	1447	1449
		1399	1394	1395
	C-O-C asymmetric stretch	1319		1328
		1313	1311	1314
				1308
	Ring breathing	1113	1108	1111
1,2,3,7,8,9-HxCDD	C=C aromatic ring skeletal vibrations		1480	
		1452	1445	1446
		1400	1391	1396
	C-O-C asymmetric stretch	1333		1328
		1314	1312	1309
			1308	
			1304	
	C-C-C tri-ring bending			1155
		1147		1143
1,2,3,6,7,8-HxCDD	C=C aromatic ring skeletal vibrations	1481		
		1455	1445	1448
		1400	1392	1395
	C-O-C asymmetric stretch	1333		1328
		1318	1310	1312
	C-C-C tri-ring bending			1150
		1147		1143
1,2,3,4,6,7,8-HpCDD	C=C aromatic ring skeletal vibrations	1480		
		1457	1451	1452
				1447
		1434	1422	1428
		1403	1392	1398
	C-O-C asymmetric stretch	1334		1329
			1297	1299
	C-C-C tri-ring bending	1148		1153
				1144
OCDD	C=C aromatic ring skeletal vibrations			1436
			1424	1429
				1419
				1402
				1399

**Table 2:** Principal FT-IR bands of PCDFs. Reprinted with permission from [19], copyright 2014.

PCDF Congener	Mode	Frequency Range (1020–1205 and 1265–1510 cm^−1^) GC/MI/FT-IR [33]	Frequency Range (1020–1205 and 1265–1510 cm^−1^) Condensed-Phase FT-IR [19]
dibenzofuran	C=C aromatic ring skeletal vibrations		1473 1465 1454 1444
	C-O-C asymmetric stretch		1320 1283
	C-C-C tri-ring bending		1189
			1196
	Ring breathing		1112
			1102
2,3,7,8-TCDF	C=C aromatic ring skeletal vibrations	1443 1402	1439 1398
	C-O-C asymmetric stretch	1289	1287
	C-H in plane deformations		1276
	C-C-C tri-ring bending		1191
	Ring breathing	1109	1107
		1099	1095
1,2,3,7,8-PeCDF	C=C aromatic ring skeletal vibrations		1460 1455 1423
		1427	1423
	C-O-C asymmetric stretch	1382 1284	1356 1301 1283 1280
	C-C-C tri-ring bending	1138	1136
	Ring breathing	1103	1100
2,3,4,7,8-PeCDF	C=C aromatic ring skeletal vibrations	1455 1429 1408	1451 1425 1404
	C-O-C asymmetric stretch	1384 1381 1287	1379 1374 1363 1285
		1162	1191 1159
			1146
	Ring breathing	1104	1101
			1088
1,2,3,4,7,8-HxCDF	C=C aromatic ring skeletal vibrations	1415	1452 1412
		1403	1399
	C-O-C asymmetric stretch	1361	1362 1357 1345
	C-H in plane deformations	1277	1276
	Ring breathing	1108	1105
		1101	1099
			1076
	C=C aromatic ring skeletal vibrations		1506
1,2,3,7,8,9-HxCDF	C-O-C asymmetric stretch	1387 1369 1361	1384 1366 1361
		1294	1296
			1289
	C-H in plane deformations	1273	1272
	C-C-C tri-ring bending	1146 1138	1143 1136
1,2,3,4,7,8,9-HpCDF	C=C aromatic ring skeletal vibrations	1405	1506 1401
	C-O-C asymmetric stretch	1381 1371 1352 1347	1376 1365 1356 1346 1282
	C-H in plane deformations	1269	1269 1266
	C-C-C tri-ring bending	1182 1142	1181 1141
	Ring breathing		1084
OCDF	C=C aromatic ring skeletal vibrations		1411 1406
	C-O-C asymmetric stretch		1364 1357 1349 1338 1328 1278
	C-H in plane		1266
	deformations		
	C-C-C tri-ring bending		1191
	Ring breathing		1109

**Table 3:** Summary of the different optical techniques described in Section 2.1.

Technique	Spectral Range cm^−1^	Molecule	Measurement Time	Frequency Accuracy (cm^−1^)	Optical Power/Wavelength	Cost	Refs
FT-IR	1020–1510	dioxins and furans	<60 s	0.9	<1 µW	Low	[19]
ECDL-QCL	1205–1310 And 1335–1590	dioxins and furans	4 min (first range); 14 min (second range)	0.1	Tens/hundreds of mW	Medium	[18]
Rapid scan QCL	860–1100	ethylene, propene, 1-butene, 2-butene, 1,3-butadiene, methanol	down to 3 ms	0.35	≤1 W	High	[56]
Dual Comb based IR spectrometer	1180–1250	Protein in Halobacterium salinarum	1 µs	0.3 *	Typically between 1 µW and 1 mW	High	[72]

* The target of this paper was fast reaction, so for different experiments the accuracy could reach the order of 3 × 10^−4^ cm^−1^ (10 MHz).

## References

[1] Breivik K., Alcock R., Li Y.-F., Bailey R.E., Fiedler H., Pacyna J.M. (2004). Primary sources of selected POPs: Regional and global scale emission inventories. Environ. Pollut..

[2] Holt E., Weber R., Stevenson G., Gaus C. (2010). Polychlorinated Dibenzo-p-Dioxins and Dibenzofurans (PCDD/Fs) Impurities in Pesticides: A Neglected Source of Contemporary Relevance. Environ. Sci. Technol..

[3] Zhao Y.-Y., Tao F.-M., Zeng E.Y. (2008). Theoretical study of the quantitative structure–activity relationships for the toxicity of dibenzo-p-dioxins. Chemosphere.

[4] Hirokawa S., Imasaka T., Imasaka T. (2005). Chlorine Substitution Pattern, Molecular Electronic Properties, and the Nature of the Ligand−Receptor Interaction:  Quantitative Property−Activity Relationships of Polychlorinated Dibenzofurans. Chem. Res. Toxicol..

[5] Larsson M., van den Berg M., Brenerová P., van Duursen M.B.M., van Ede K.I., Lohr C., Luecke-Johansson S., Machala M., Neser S., Pěnčíková K. (2015). Consensus Toxicity Factors for Polychlorinated Dibenzo-p-dioxins, Dibenzofurans, and Biphenyls Combining in Silico Models and Extensive in Vitro Screening of AhR-Mediated Effects in Human and Rodent Cells. Chem. Res. Toxicol..

[6] Mimura J., Fujii-Kuriyama Y. (2003). Functional role of AhR in the expression of toxic effects by TCDD. Biochim. Biophys. Acta Gen. Subj..

[7] Van den Berg M., Birnbaum L.S., Denison M., de Vito M., Farland W., Feeley M., Fiedler H., Hakansson H., Hanberg A., Haws L. (2006). The 2005 World Health Organization Re-evaluation of Human and Mammalian Toxic Equivalency Factors for Dioxins and Dioxin-like Compounds. Toxicol. Sci..

[8] Schug T.T., Johnson A.F., Birnbaum L.S., Colborn T., Guillette J.L.J., Crews D.P., Collins T., Soto A.M., vom Saal F.S., McLachlan J.A. (2016). Minireview: Endocrine Disruptors: Past Lessons and Future Directions. Mol. Endocrinol..

[9] Mrema E.J., Rubino F.M., Brambilla G., Moretto A., Tsatsakis A.M., Colosio C. (2013). Persistent organochlorinated pesticides and mechanisms of their toxicity. Toxicology.

[10] Patrizi B., Siciliani de Cumis M. (2018). TCDD Toxicity Mediated by Epigenetic Mechanisms. Int. J. Mol. Sci..

[11] Esser C., Rannug A. (2015). The Aryl Hydrocarbon Receptor in Barrier Organ Physiology, Immunology, and Toxicology. Pharmacol. Rev..

[12] Pirkle J.L., Wolfe W.H., Patterson D.G., Needham L.L., Michalek J.E., Miner J.C., Peterson M.R., Phillips D.L. (1989). Estimates of the half-life of 2,3,7,8-tetrachlorodibenzo-p-dioxin in Vietnam veterans of operation ranch hand. J. Toxicol. Environ. Health.

[13] Wolfe W.H., Michalek J.E., Miner J.C., Pirkle J.L., Caudill S.P., Patterson D.G., Needham L.L. (1994). Determinants of TCDD half-life in veterans of operation ranch hand. J. Toxicol. Environ. Health.

[14] Safe S. (1990). Polychlorinated Biphenyls (PCBs), Dibenzo-p-Dioxins (PCDDs), Dibenzofurans (PCDFs), and Related Compounds: Environmental and Mechanistic Considerations Which Support the Development of Toxic Equivalency Factors (TEFs). Crit. Rev. Toxicol..

[15] Hennig B., Meerarani P., Slim R., Toborek M., Daugherty A., Silverstone A.E., Robertson L.W. (2002). Proinflammatory Properties of Coplanar PCBs: In Vitro and in Vivo Evidence. Toxicol. Appl. Pharmacol..

[16] Carpenter D.O. (2006). Polychlorinated biphenyls (PCBs): Routes of exposure and effects on human health. Rev. Environ. Health.

[17] Gullett B.K., Oudejans L., Tabor D., Touati A., Ryan S. (2012). Near-Real-Time Combustion Monitoring for PCDD/PCDF Indicators by GC-REMPI-TOFMS. Environ. Sci. Technol..

[18] De Cumis M.S., D’Amato F., Viciani S., Patrizi B., Foggi P., Galea C.L. (2013). First quantitative measurements by IR spectroscopy of dioxins and furans by means of broadly tunable quantum cascade lasers. Laser Phys..

[19] Patrizi B., de Cumis M.S., Viciani S., D’Amato F., Foggi P. (2014). Characteristic vibrational frequencies of toxic polychlorinated dibenzo-dioxins and -furans. J. Hazard. Mater..

[20] Deguchi Y., Dobashi S., Fukuda N., Shinoda K., Morita M. (2003). Real-Time PCB Monitoring Using Time-of-Flight Mass Spectrometry with Picosecond Laser Ionization. Environ. Sci. Technol..

[21] Wang Z.Z., Deguchi Y., Yan J.J., Liu J.P. (2015). Breakdown Pattern of Hydrocarbons by Laser Breakdown Time-of-flight Mass Spectrometry. Spectrosc. Lett..

[22] Ncube S., Madikizela L., Cukrowska E., Chimuka L. (2018). Recent advances in the adsorbents for isolation of polycyclic aromatic hydrocarbons (PAHs) from environmental sample solutions. TrAC Trends Anal. Chem..

[23] Patil S.S., Shedbalkar U.U., Truskewycz A., Chopade B.A., Ball A.S. (2016). Nanoparticles for environmental clean-up: A review of potential risks and emerging solutions. Environ. Technol. Innov..

[24] Carabias-Martínez R., Rodríguez-Gonzalo E., Revilla-Ruiz P., Hernández-Méndez I. (2005). Pressurized liquid extraction in the analysis of food and biological samples. J. Chroma A.

[25] Focant J.-F., Pirard C., de Pauw E. (2004). Automated sample preparation-fractionation for the measurement of dioxins and related compounds in biological matrices: A review. Talanta.

[26] Dimpe K.M., Nomngongo P.N. (2016). Current sample preparation methodologies for analysis of emerging pollutants in different environmental matrices. TrAC. Trends Anal. Chem..

[27] Hoff R.B., Pizzolato T.M. (2018). Combining extraction and purification steps in sample preparation for environmental matrices: A review of matrix solid phase dispersion (MSPD) and pressurized liquid extraction (PLE) applications. TrAC Trends Anal. Chem..

[28] Zheng Y., Cai D., Huang B., Han J., Chen Q., Zhang J., Zhang J., Wang X., Shen H. (2019). Simultaneous detection of multiple hydroxylated polychlorinated biphenyls from biological samples using ultra-high-performance liquid chromatography with isotope dilution tandem mass spectrometry. J. Sep. Sci..

[29] Petrovic M., Eljarrat E., de Alda M.J.L., Barceló D. (2002). Recent advances in the mass spectrometric analysis related to endocrine disrupting compounds in aquatic environmental samples. J. Chromatogr. A.

[30] Lorenzo M., Pico Y., Schrenk D., Cartus A. (2017). Gas Chromatography and Mass Spectroscopy Techniques for the Detection of Chemical Contaminants and Residues in Foods. Chemical Contaminants and Residues in Food.

[31] Pizzini S., Piazza R., Cozzi G., Barbante C. (2016). Simultaneous determination of halogenated contaminants and polycyclic aromatic hydrocarbons: A multi-analyte method applied to filter-feeding edible organisms. Anal. Bioanal. Chem..

[32] Smith B. (2011). Fundamentals of Fourier Transform Infrared Spectroscopy.

[33] Wurrey C.J., Fairless B.J., Kimball H.E. (1989). Gas Chromatographic/Matrix Isolation/Fourier Transform Infrared Spectra of the Laterally Chlorinated Dibenzo-p-Dioxins and Dibenzofurans. Appl. Spectrosc..

[34] Grainger J., Gelbaum L.T. (1987). Tetrachlorodibenzo-p-Dioxin Isomer Differentiation by Capillary Gas Chromatography Fourier Transform-Infrared Spectroscopy. Appl. Spectrosc..

[35] Coddington I., Newbury N., Swann W. (2016). Dual-comb spectroscopy. Optica.

[36] Hugi A., Villares G., Blaser S., Liu H.C., Faist J. (2012). Mid-infrared frequency comb based on a quantum cascade laser. Nature.

[37] Reichert J., Holzwarth R., Udem T., Hänsch T.W. (1999). Measuring the frequency of light with mode-locked lasers. Opt. Commun..

[38] Giordmaine J.A., Miller R.C. (1965). Tunable Coherent Parametric Oscillation in LiNbO_3_ at Optical Frequencies. Phys. Rev. Lett..

[39] Faist J., Capasso F., Sivco D.L., Sirtori C., Hutchinson A.L., Cho A.Y. (1994). Quantum Cascade Laser. Science.

[40] Vurgaftman I., Weih R., Kamp M., Meyer J.R., Canedy C.L., Kim C.S., Kim M., Bewley W.W., Merritt C.D., Abell J. (2015). Interband cascade lasers. J. Phys. D Appl. Phys..

[41] Vasilyev S., Moskalev I.S., Smolski V.O., Peppers J.M., Mirov M., Muraviev A.V., Zawilski K., Schunemann P.G., Mirov S.B., Vodopyanov K.L. (2019). Super-octave longwave mid-infrared coherent transients produced by optical rectification of few-cycle 2.5-μm pulses. Optica.

[42] Young C., Kim S.S., Luzinova Y., Weida M., Arnone D., Takeuchi E., Day T., Mizaikoff B. (2009). External cavity widely tunable quantum cascade laser based hollow waveguide gas sensors for multianalyte detection. Sens. Actuators B Chem..

[43] Hodgkinson J., Tatam R.P. (2012). Optical gas sensing: A review. Meas. Sci. Technol..

[44] Kosterev A., Wysocki G., Bakhirkin Y., So S., Lewicki R., Fraser M., Tittel F., Curl R.F. (2008). Application of quantum cascade lasers to trace gas analysis. Appl. Phys. B.

[45] De Cumis M.S., Viciani S., Galli I., Mazzotti D., Sorci F., Severi M., D’Amato F. (2015). Note: An analyzer for field detection of H2S by using cavity ring-down at 1.57 μm. Rev. Sci. Instrum..

[46] Chiarugi A., Viciani S., D’Amato F., Burton M. (2018). Diode laser-based gas analyser for the simultaneous measurement of CO_2_ and HF in volcanic plumes. Atmos. Meas. Tech..

[47] Viciani S., Montori A., Chiarugi A., D’Amato F. (2018). A Portable Quantum Cascade Laser Spectrometer for Atmospheric Measurements of Carbon Monoxide. Sensors.

[48] Patrizi B., Lapini A., di Donato M., Marcelli A., Lima M., Righini R., Foggi P., Baiocco P., Bonamore A., Boffi A. (2014). Role of Local Structure and Dynamics of Small Ligand Migration in Proteins: A Study of a Mutated Truncated Hemoprotein from Thermobifida fusca by Time Resolved MIR Spectroscopy. J. Phys. Chem. B.

[49] Lapini A., di Donato M., Patrizi B., Marcelli A., Lima M., Righini R., Foggi P., Sciamanna N., Boffi A. (2012). Carbon Monoxide Recombination Dynamics in Truncated Hemoglobins Studied with Visible-Pump MidIR-Probe Spectroscopy. J. Phys. Chem. B.

[50] Hugi A., Terazzi R., Bonetti Y., Wittmann A., Fischer M., Beck M., Faist J., Gini E. (2009). External cavity quantum cascade laser tunable from 7.6 to 11.4 μm. Appl. Phys. Lett..

[51] Brandstetter M., Lendl B. (2012). Tunable mid-infrared lasers in physical chemosensors towards the detection of physiologically relevant parameters in biofluids. Sens. Actuators B Chem..

[52] Da Silveira Petruci J.F., Fortes P.R., Kokoric V., Wilk A., Raimundo I.M., Cardoso A.A., Mizaikoff B. (2013). Real-time monitoring of ozone in air using substrate-integrated hollow waveguide mid-infrared sensors. Sci. Rep..

[53] Jouy P., Bonetti Y., Hans K., Gianella M., Sigrist M., Mangold M., Tuzson B., Emmenegger L., Wägli P., Homsy A. (2013). Multi-Wavelength QCL Based MIR Spectroscopy for Fluids and Gases, CLEO: 2013, San Jose, California, 2013/06/09.

[54] Lyakh A., Barron-Jimenez R., Dunayevskiy I., Go R., Tsvid E., Patel K.C. (2016). Progress in Rapidly-Tunable External Cavity Quantum Cascade Lasers with a Frequency-Shifted Feedback. Photonics.

[55] Maulini R., Beck M., Faist J., Gini E. (2004). Broadband tuning of external cavity bound-to-continuum quantum-cascade lasers. Appl. Phys. Lett..

[56] Strand C.L., Ding Y., Johnson S.E., Hanson R.K. (2019). Measurement of the mid-infrared absorption spectra of ethylene (C2H4) and other molecules at high temperatures and pressures. J. Quant. Spectrosc. Radiat. Transfer.

[57] Brandstetter M., Genner A., Anic K., Lendl B. (2010). Tunable external cavity quantum cascade laser for the simultaneous determination of glucose and lactate in aqueous phase. Analyst.

[58] Alcaráz M.R., Schwaighofer A., Kristament C., Ramer G., Brandstetter M., Goicoechea H., Lendl B. (2015). External-Cavity Quantum Cascade Laser Spectroscopy for Mid-IR Transmission Measurements of Proteins in Aqueous Solution. Anal. Chem..

[59] Montalvo G., Waegele M.M., Shandler S., Gai F., DeGrado W.F. (2010). Infrared Signature and Folding Dynamics of a Helical β-Peptide. J. Am. Chem. Soc..

[60] Wörle K., Seichter F., Wilk A., Armacost C., Day T., Godejohann M., Wachter U., Vogt J., Radermacher P., Mizaikoff B. (2013). Breath Analysis with Broadly Tunable Quantum Cascade Lasers. Anal. Chem..

[61] Waegele M.M., Gai F. (2010). Infrared Study of the Folding Mechanism of a Helical Hairpin: Porcine PYY. Biochemistry.

[62] Furstenberg R., Kendziora C.A., Papantonakis M.R., Nguyen V., McGill R.A. (2012). Chemical imaging using infrared photothermal microspectroscopy. SPIE.

[63] Ravaro M., Locatelli M., Pugliese E., di Leo I., de Cumis M.S., D’Amato F., Poggi P., Consolino L., Meucci R., Ferraro P. (2014). Mid-infrared digital holography and holographic interferometry with a tunable quantum cascade laser. Opt. Lett..

[64] Pleitez M.A., Lieblein T., Bauer A., Hertzberg O., von Lilienfeld-Toal H., Mäntele W. (2013). In Vivo Noninvasive Monitoring of Glucose Concentration in Human Epidermis by Mid-Infrared Pulsed Photoacoustic Spectroscopy. Anal. Chem..

[65] Pilling M.J., Henderson A., Gardner P. (2017). Quantum Cascade Laser Spectral Histopathology: Breast Cancer Diagnostics Using High Throughput Chemical Imaging. Anal. Chem..

[66] Yeh K., Schulmerich M., Bhargava R. (2013). Mid-infrared microspectroscopic imaging with a quantum cascade laser. SPIE.

[67] Adler F., Thorpe M.J., Cossel K.C., Ye J. (2010). Cavity-Enhanced Direct Frequency Comb Spectroscopy: Technology and Applications. Annu. Rev. Anal. Chem..

[68] Picqué N., Hänsch T.W. (2019). Frequency comb spectroscopy. Nat. Photonics.

[69] De Cumis M.S., Eramo R., Coluccelli N., Galzerano G., Laporta P., Pastor P.C. (2018). Multiplexed direct-frequency-comb Vernier spectroscopy of carbon dioxide 2ν_1_ + ν_3_ ro-vibrational combination band. J. Chem. Phys..

[70] Villares G., Hugi A., Blaser S., Faist J. (2014). Dual-comb spectroscopy based on quantum-cascade-laser frequency combs. Nat. Commun..

[71] Bagheri M., Frez C., Sterczewski L.A., Gruidin I., Fradet M., Vurgaftman I., Canedy C.L., Bewley W.W., Merritt C.D., Kim C.S. (2018). Passively mode-locked interband cascade optical frequency combs. Sci. Rep..

[72] Klocke J.L., Mangold M., Allmendinger P., Hugi A., Geiser M., Jouy P., Faist J., Kottke T. (2018). Single-Shot Sub-microsecond Mid-infrared Spectroscopy on Protein Reactions with Quantum Cascade Laser Frequency Combs. Anal. Chem..

[73] Taylor A.B., Zijlstra P. (2017). Single-Molecule Plasmon Sensing: Current Status and Future Prospects. ACS Sens..

[74] Giorgini A., Avino S., Malara P., Zullo R., De Natale P., Mrkvová K., Homola J., Gagliardi G. (2018). Surface-plasmon optical-heterodyne clock biosensor. Sens. Actuators B Chem..

[75] Greive S.J., Weitzel S.E., Goodarzi J.P., Main L.J., Pasman Z., von Hippel P.H. (2008). Monitoring RNA transcription in real time by using surface plasmon resonance. Proc. Natl. Acad. Sci. USA.

[76] Bocková M., Chadtová Song X., Gedeonová E., Levová K., Kalousová M., Zima T., Homola J. (2016). Surface plasmon resonance biosensor for detection of pregnancy associated plasma protein A2 in clinical samples. Anal. Bioanal. Chem..

[77] Kabashin A.V., Patskovsky S., Grigorenko A.N. (2009). Phase and amplitude sensitivities in surface plasmon resonance bio and chemical sensing. Opt. Express.

[78] Gangopadhyay T.K., Giorgini A., Halder A., Pal M., Paul M.C., Avino S., Gagliardi G. (2015). Detection of chemicals using a novel fiber-optic sensor element built in fiber loop ring-resonators. Sens. Actuators B Chem..

[79] Gagliardi G. (2014). Cavity-Enhanced Spectroscopy and Sensing.

[80] Soh N., Tokuda T., Watanabe T., Mishima K., Imato T., Masadome T., Asano Y., Okutani S., Niwa O., Brown S. (2003). A surface plasmon resonance immunosensor for detecting a dioxin precursor using a gold binding polypeptide. Talanta.

[81] Shimomura M., Nomura Y., Zhang W., Sakino M., Lee K.-H., Ikebukuro K., Karube I. (2001). Simple and rapid detection method using surface plasmon resonance for dioxins, polychlorinated biphenylx and atrazine. Anal. Chim. Acta.

[82] Abalde-Cela S., Ho S., Rodríguez-González B., Correa-Duarte M.A., Álvarez-Puebla R.A., Liz-Marzán L.M., Kotov N.A. (2009). Loading of Exponentially Grown LBL Films with Silver Nanoparticles and Their Application to Generalized SERS Detection. Angew. Chem. Int. Ed..

[83] Yang Y., Meng G. (2010). Ag dendritic nanostructures for rapid detection of polychlorinated biphenyls based on surface-enhanced Raman scattering effect. J. Appl. Phys..

[84] Bantz K.C., Haynes C.L. (2009). Surface-enhanced Raman scattering detection and discrimination of polychlorinated biphenyls. Vib. Spectrosc..

[85] Zhu C., Meng G., Huang Q., Huang Z. (2012). Vertically aligned Ag nanoplate-assembled film as a sensitive and reproducible SERS substrate for the detection of PCB-77. J. Hazard. Mater..

[86] Fleischmann M., Hendra P.J., McQuillan A.J. (1974). Raman spectra of pyridine adsorbed at a silver electrode. Chem. Phys. Lett..

[87] Stiles P.L., Dieringer J.A., Shah N.C., van Duyne R.P. (2008). Surface-Enhanced Raman Spectroscopy. Annu. Rev. Anal. Chem..

[88] Wang M., Meng G., Huang Q., Li M., Li Z., Tang C. (2011). Fluorescence detection of trace PCB101 based on PITC immobilized on porous AAO membrane. Analyst.

[89] Li M., Meng G., Huang Q., Yin Z., Wu M., Zhang Z., Kong M. (2010). Prototype of a Porous ZnO SPV-Based Sensor for PCB Detection at Room Temperature under Visible Light Illumination. Langmuir.

[90] Tang J., Chen W., Ju H. (2019). Rapid detection of pesticide residues using a silver nanoparticles coated glass bead as nonplanar substrate for SERS sensing. Sens. Actuators B Chem..

[91] Schädle T., Mizaikoff B. (2016). Mid-Infrared Waveguides: A Perspective. Appl. Spectros..

[92] Chen Y., Lin H., Hu J., Li M. (2014). Heterogeneously Integrated Silicon Photonics for the Mid-Infrared and Spectroscopic Sensing. ACS Nano.

